# Prediction of Pituitary Adenoma’s Volumetric Response to Gamma Knife Radiosurgery Using Machine Learning-Supported MRI Radiomics

**DOI:** 10.3390/jcm14092896

**Published:** 2025-04-23

**Authors:** Herwin Speckter, Marko Radulovic, Erwin Lazo, Giancarlo Hernandez, Jose Bido, Diones Rivera, Luis Suazo, Santiago Valenzuela, Peter Stoeter, Velicko Vranes

**Affiliations:** 1 Centro Gamma Knife Dominicano, CEDIMAT, Plaza de la Salud, Santo Domingo 10514, Dominican Republic; 2 Department of Radiology, CEDIMAT, Plaza de la Salud, Santo Domingo 10514, Dominican Republic; 3 Department of Basic and Environmental Sciences, Instituto Tecnológico de Santo Domingo (INTEC), Santo Domingo 10602, Dominican Republic; velicko.vranes@intec.edu.do; 4 Department of Experimental Oncology, Institute for Oncology & Radiology of Serbia, 11 000 Belgrade, Serbia; 5 School of Medicine, Universidad Pedro Henriquez Urena (UNPHU), Santo Domingo 10203, Dominican Republic

**Keywords:** pituitary adenoma, gamma knife radiosurgery, radiomics, outcome prediction, machine learning

## Abstract

**Background/Objectives:** Gamma knife radiosurgery (GKRS) is widely performed as an adjuvant management of patients with residual or recurrent pituitary adenoma (PA). However, the variability in the tumor volume response to GKRS emphasizes the need for reliable predictors of treatment outcomes. The application of radiomics, an analytical approach for quantitative imaging, remains unexplored in predicting treatment responses for PAs. This study aimed to pioneer the use of radiomic MRI analysis to predict the volumetric response of PA to GKRS. **Methods:** This retrospective observational cohort study involved 81 patients who underwent GKRS for PA. Pre-treatment 3-Tesla MRI scans were used to extract radiomic features capturing the intensity, shape, and texture of the tumors. Radiomic signatures were generated using the least absolute shrinkage and selection operator (LASSO) for feature selection, in conjunction with several classifiers: random forest, naïve Bayes, kNN, logistic regression, neural network, and SVM. **Results:** The models demonstrated predictive performance in the test folds, with AUC values ranging from 0.759 to 0.928 and R^2^ values between 0.272 and 0.665. Single-sequence T1w, dual-sequence T1w + CE-T1w, and multi-modality including clinicopathological (CP) parameters (CP + T1w + CE-T1w) achieved rather similar prognostic performance in the test folds, with respective AUCs of 0.928, 0.899, and 0.909. All these radiomics models significantly outperformed a benchmark model involving only CP features (AUC = 0.846). **Conclusions:** This study represents a radiomic analysis focused on predicting the volume response of PAs to GKRS to facilitate treatment individualization. The developed MRI-based radiomics models exhibited superior classification performance compared with the benchmark model composed solely of standard clinicopathological parameters.

## 1. Introduction

Pituitary adenomas (PAs) are predominantly benign brain tumors, constituting 10–20% of all intracranial neoplasms, and account for most sellar and parasellar tumors. Gamma knife radiosurgery (GKRS) has emerged as an effective and minimally invasive treatment modality with high therapeutic efficacy for various intracranial pathologies, including nonfunctioning and functioning PAs. Radiosurgery (SRS), involving hypofractionated SRS delivered in up to five fractions, achieves tumor control rates ranging from 80% to over 90% in published reports [1,2,3]. Although GKRS achieves favorable outcomes, the individual treatment response varies substantially among patients, necessitating more reliable predictors of individual treatment outcomes [4,5,6].

A common approach in PA treatment is the trans-sphenoidal removal of the central part of the PA, frequently leaving the remnants within the cavernous sinus for GKRS treatment. The volume response, commonly assessed through post-treatment imaging, is an essential indicator of GKRS’s efficacy and can inform subsequent management decisions. Identifying reliable predictors of volume response would enable early intervention for poor and non-responders, potentially improving overall patient outcomes. If a reliable pre-treatment outcome prediction existed, surgical treatment could be avoided in selected PAs predicted to respond significantly to GKRS. Conversely, in cases with a less favorable prognosis for GKRS, where higher radiation doses may be required, upfront surgical removal of the central parts of the PA may be preferable to avoid radiation injury to the adjacent critical structures, particularly the optic nerves, chiasm, and tracts.

Radiomics focuses on extracting and analyzing the intensity, morphological, and texture features of medical images. These features capture complex tumor characteristics that are often invisible to the human eye, enabling the potential identification of imaging biomarkers for treatment response prediction. Radiomics has shown promise in various oncological contexts, allowing for non-invasive prediction of treatment response or prognosis of disease outcome [7,8].

We hypothesized that radiomics prediction signatures could outperform the benchmark signature relying only on routine clinicopathological (CP) features for predicting PA’s volumetric response.

The objective of this study was to develop several single-modality and multi-modality radiomic prediction signatures, both with and without CP parameters, to predict the volumetric response of PAs to GKRS, using pre-treatment MRI. Additionally, the study aimed to compare the predictive performance of these signatures in the test folds. Improving the prediction of treatment outcomes is highly clinically relevant because it enables personalized treatment planning, optimizes therapeutic strategies, and thereby enhances patient care.

## 2. Methods

This study adheres to the guidelines set out in the STROBE statement for cohort studies.

### 2.1. Ethics Approval Statement

The study received approval (CEI-391, 24 April 2019) from our Institutional Review Board and adheres to The Code of Ethics of the World Medical Association (Declaration of Helsinki), as published in the *British Medical Journal* (18 July 1964) and its 7th revised edition in 2013. The need for written informed consent was waived by the ethics committee due to the retrospective nature of the analysis.

### 2.2. Patients

Included were 81 patients between 11.4 and 83.2 years of age (mean: 45.6) with imaging-diagnosed PA treated at our gamma knife center. Of the 81 PAs, 52 were nonfunctional PAs, while 29 were functional PAs, including 5 growth hormone (GH)-secreting adenomas, 7 adrenocorticotropic hormone (ACTH)-secreting adenomas, and 17 prolactin hormone (PRL)-secreting adenomas. 15 PAs were treated upfront, 45 PAs had one surgery before SRS, 16 PAs had two surgeries, and 5 PAs were operated on 3 times before SRS. MRI had been performed within four weeks before radiosurgery, with available follow-up data after an interval of six months or longer (range: 6.7–105.5 months, mean: 40.4; Table 1). Tumor volumes ranged between 0.18 and 40.33 cm^3^ (mean: 6.30).

### 2.3. Sample Size Calculation

The prospective sample size calculation was based on a pilot experiment with 30 patients and required 36 patients for alpha = 0.05, beta = 0.20, and AUC = 0.76 (Medcalc 14.8.1; MedCalc Software Ltd., Ostend, Belgium). These 30 patients were included in the final cohort. The actual AUCs obtained for the calculated scores integrating radiomics and CP features ranged between 0.759 and 0.928, with a final sample size of 81 patients in T1w, 81 in CE-T1w, 48 in T2w, and 41 in FLAIR.

### 2.4. Gamma Knife Treatment

The gamma knife technique was previously described [7]. On the day of the treatment, after placing a stereotactic G frame (Elekta AB), under sedation and local anesthesia, contrast-enhanced 3D computed tomography (CT) imaging was obtained. Pre-treatment MRI sequences, acquired less than four weeks before, were then coregistered to the stereotactic CT. Treatments were planned on a Leksell GammaPlan 10.1 workstation (Elekta Instrument AB, Stockholm, Sweden), carefully respecting the dose constraints, particularly of the optic apparatus.

The margin dose varied from 12 to 40 Gy (mean: 20.5 Gy), depending on the tumor’s size, location, and hormone status (Table 1). Forty-nine PAs were treated in a single session, with margin doses ranging from 12 to 35 Gy (mean: 17.8 Gy). According to our institutional protocol, lesions abutting the anterior optic pathway are treated using hypofractionated radiosurgery (HFSRS). Thirty-two PAs were treated with 5.0 to 9.0 Gy (mean: 6.1 Gy) for 3 to 5 days (mean: 4.03). The biologically effective dose (BED) is routinely used to compare doses of different dose–fraction regimens relying on the widely accepted linear quadratic model, with its known limitations for high doses [9]. HFSRS doses can be converted to single-fraction equivalent doses (SFED) to intuitively compare radiation effects with conventional physical doses [10]. Margin SFED varied from 11.1 to 35.0 Gy (mean: 16.2 Gy), applying an α/β ratio of 2.47 Gy for nonfunctional PAs or 4.91 Gy for functioning Pas [11,12].

### 2.5. MRI

MRI was performed on a 3-Tesla scanner (Achieva; Philips, Eindhoven, Netherlands). Three-dimensional T1-weighted non-contrast (T1w), contrast-enhanced (CE-T1w), T2w, and FLAIR sequences were acquired with the following sequence parameters.

1. Three-dimensional T1w magnetization-prepared rapid acquisition (MPRAGE) sequence: gradient echo; TR/TE/TI, 6.8/3.2/900 ms; flip angle, 8°; measured voxel size, 0.6 × 0.6 × 1.0 mm, before and after intravenous injection of contrast medium.
2. T2w sequence: TR/TE 3693.8/80 ms; 150 transversal slices; thickness, 1 mm; matrix, 512 × 512.
3. Fluid-attenuated inversion recovery (FLAIR) sequence: TR/TE/TI, 11,000/120/2800 ms; 90 transversal slices; thickness, 2 mm; matrix, 512 × 512.

### 2.6. Postprocessing

In all 81 patients, PA volumes were delineated and measured from CE-T1w images on the Leksell GammaPlan workstation. MRI sequences were coregistered to the stereotactic 3D contrast-enhanced CT acquired on the treatment day. Image sets were verified for high image quality. Sequences with artifacts were excluded.

### 2.7. Follow-Up

Imaging and clinical follow-up were performed every six months for the first two years after GKRS and annually thereafter.

### 2.8. Feature Extraction

The radiomics analysis was conducted using the open-source Pyradiomics plugin, integrated into 3D Slicer (version 5.6.1) [13]. The Pyradiomics parameter file informed the computation of all available image transformations and feature types, resulting in a total of 2156 features per MRI scan.

Feature extraction was performed on the original images (107 features) and on images transformed by wavelet, square, square root, logarithm, gradient, exponential, Laplacian of Gaussian (LoG), and Local Binary Pattern (LBP 2D) filters. For detailed descriptions of the extracted radiomics features, please see: https://pyradiomics.readthedocs.io/en/latest/features.html (accessed on 19 April 2025).

### 2.9. Model Selection

Features were pre-selected by discarding those without a significant correlation with the PA’s volume change outcome, based on Pearson’s correlation test (Statistica 12.0, StatSoft, Hamburg, Germany). Predictive models were constructed using the features selected by LASSO regression with integrated 10-fold cross-validation and the following classifiers: random forest, naïve Bayes, kNN, logistic regression, neural network, and SVM (Orange Data Mining, University of Ljubljana, Slovenia).

### 2.10. Evaluation of Predictive Performance

The PA’s volume change per natural logarithm of time was chosen as the endpoint to account for the near-exponential decrease in tumor volume over time [14]. Beyond an initial 6-month phase, our data indicate that most PA volumes follow an exponential trend. To compare volume changes during long FU periods (FUP), tumor volume change per natural logarithm of time is more accurate than volume change per time. This was calculated as the difference in tumor volume before SRS and at the last follow-up, divided by the initial volume and the natural logarithm of the time since SRS to the last FU [14]:

$$\mathrm{Relative}\;\mathrm{volume}\;\mathrm{change}\;\mathrm{per}\;\ln\left(\mathrm{month}\right)=\frac{\mathrm{Volume}\;\mathrm{at}\;\mathrm{last}\;\mathrm{FUP}-\mathrm{Initial}\;\mathrm{volume}\;\mathrm{at}\;\mathrm{SRS}}{\mathrm{Initial}\;\mathrm{Volume}\times \ln\left(\mathrm{FUP}\right)}$$

Predictive performance was assessed using ROC analysis (IBM SPSS v28, Armonk, NY, USA; Orange Data Mining), with statistical analyses considering both continuous independent variables and continuous or categorized dependent outcomes.

### 2.11. Validation

Validation involved bootstrapping for ROC analysis and split-sample cross-validation via LASSO. Besides LASSO cross-validation in the training folds (51 patients), models were validated on eight random test folds (30 patients).

## 3. Results

### 3.1. Patients’ Characteristics

The treatment results are presented in Table 1. After a mean FUP of 40.4 months, the control rate was 98.8%. As there are no standardized radiographic criteria for assessing PAs’ treatment response, we used the RANO criteria [15] stated by Imber et al. for PA response characterization [16]. Partial response was achieved in 60 (74.1%) PAs, with stable disease found in 20 (24.7%) patients, while 1 (1.2%) progressive PA was observed at last follow-up. The volumetric outcome was notably better in functioning PAs, with a volume reduction of 2.21% per month, compared with a lower volume reduction per month of 1.22% for nonfunctional PAs. This difference in volumetric response is probably attributed to the significantly higher doses used in treating functioning PAs, which are required to obtain hormone remission.

### 3.2. Experimental Design

**Figure 1:**
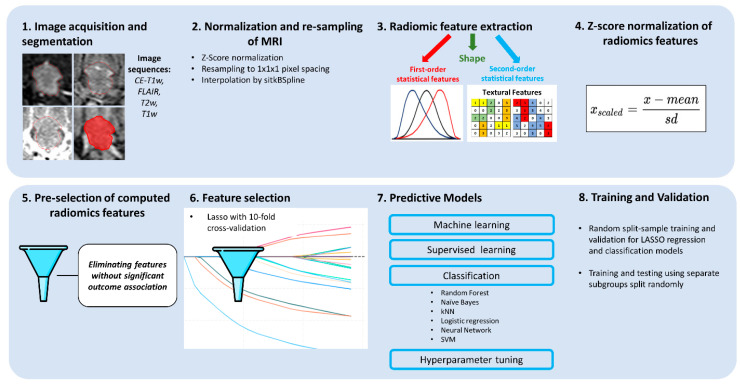
Workflow of radiomics analysis. MRIs were acquired before GKRS in four sequences and tumor VOIs were segmented. Before radiomics analysis, images underwent z-score normalization, resampling, and interpolation. All feature classes and image transformations produced by all available filters were calculated, and the feature values were further normalized by the z-score. Features lacking a significant association with the outcome were discarded. LASSO was used for further feature selection, followed by machine learning classifiers to train a prognostic model and evaluate its generalizability in the test folds.

The workflow of the study is shown in Figure 1. Models were developed for individual modalities (CP, T1w, CE-T1w, T2w, FLAIR) and combined modalities (T1w + CE-T1w, CP + T1w + CE-T1w), focusing on the T1w and CE-T1w MRI sequences available for all 81 patients. This facilitated multi-sequence model construction and validation across eight test folds. Tumor volume response prediction used R^2^ from linear LASSO (Table 2) for continuous outcomes, and logit LASSO with six classifiers for categorized outcomes, using AUC and accuracy for evaluation (Table 3 and Table 4).

### 3.3. The Predictive Models for PA’s Response to Radiosurgery

The constructed predictive models were initially tested against the continuous, uncategorized tumor volume response outcomes (Table 2). Thereby, T2w showed the best association with the outcomes (R^2^ = 0.665), followed by the most comprehensive models: CP + T1w + CE-T1w (R^2^ = 0.584) and T1w + CE-T1w (R^2^ = 0.502). The optimal performance of T2w was notable, but because of its availability in a smaller number of patients, this result was interpreted as preliminary. Our focus was thus directed towards CP + T1w + CE-T1w, identified as the second-best performer (Table 2).

**Figure 2:**
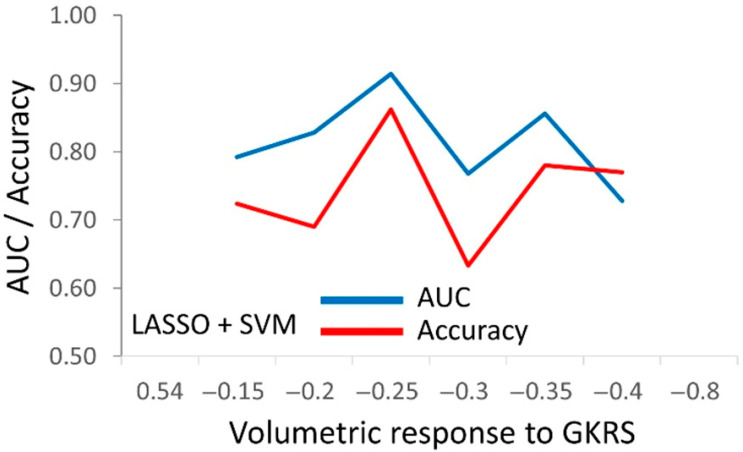
The ROC analysis of the comprehensive prognostic score of CP + T1w + CE-T1w against the outcome categorized by the six indicated cutpoints. This score includes the CP features and radiomics features combined from two MRI sequences. The tumor volume response ranged between −0.80% and +0.54% per month (*x*-axis). Using logit LASSO, we selected eight features for classification by random forest, naïve Bayes, kNN, logistic regression, neural networks, and SVM. The AUC (blue line) and accuracy (orange line) values on the *y*-axis signify the optimal prognostic performance achieved at each cutpoint by SVM, which provided the optimal classification. Increasing values of the CP + T1w + CE-T1w score were associated a with lower tumor volume response to GKRS.

The optimal cutpoints for the comprehensive model, combining CP and radiomics features (CP + T1w + CE-T1w), were identified by testing against six different cutpoints in the outcome (Figure 2). The cutpoint of a −0.25% tumor volumetric response per natural logarithm of time was identified as the most suitable for prognosis using the available CP and radiomics features (Figure 2).

After determining the optimal cutpoint for outcome categorization, the predictive performance of individual CP parameters was evaluated using the *t*-test for continuous and the Chi-square test for binary values (Table 3). Age, fraction number, accumulated dose, SFED, and hormone secretion status showed significant associations with the −0.25% outcome (Table 3). Models using only CP or CE-T1w features had the weakest link with the −0.25% outcome, while those with T1w features performed best. Notably, there was no significant difference in predictive performance among the T1w, T1w + CE-T1w, and CP + T1w + CE-T1w models, as confirmed by an independent sample *t*-test. To reduce the feature burden on LASSO, features were pre-selected on the basis of their Pearson correlation with the outcome cutpoint at −0.25%, enhancing the models’ prognostic performance. These features were refined through L1 LASSO selection, with the top eight used in classification by random forest, naïve Bayes, kNN, logistic regression, neural networks, and SVM classifiers (Table 4). Comparing the classifiers’ performance using the CP + T1w + CE-T1w model for the −0.25% outcome cutpoint revealed that logistic regression, the neural network, and SVM showed similar classification performance in the test folds, with random forest, naïve Bayes, and kNN performing less effectively (Table 4).

Table 5 details selected features and coefficients for the model incorporating CP, T1w, and CE-T1w features, highlighting the inclusion of only one CE-T1w feature, likely due to its inferior predictive performance (Table 5).

## 4. Discussion

This initial study demonstrates that radiomic signatures built on pre-GKRS MRI data can be used to predict tumors’ volumetric response to radiosurgery. The computed radiomic models outperformed the benchmark model, which included only clinicopathological features. The T1w and T2w models were the best predictive performers, while the dual sequence T1w + CE-T1w and the merged CP + T1w + T2w models did not provide a statistically significant improvement over the single-modality models, as judged by the *t*-test. All models, except for T2w and FLAIR (due to their low numbers), underwent additional evaluation for generalizability using cross-validation. The results demonstrated that the predictive performance in the training folds was largely retained in unseen test folds, with the remaining AUCs consistently exceeding 0.90.

Radiomics analysis treats MRI as minable data by extracting quantitative computational features. It gains importance as clinical imaging becomes increasingly widespread. To the best of our knowledge, no prior studies have employed texture or radiomics analyses on pituitary adenomas. Our group and others have performed texture analyses on meningiomas [17], vestibular schwannomas [18,19,20], and brain metastases [21,22]. In a previous study investigating the use of radiomics features to develop a treatment prognosis following SRS, our group applied radiomics to pre-radiosurgical MRI to predict the long-term outcomes of WHO Grade 1 meningiomas after SRS, achieving satisfactory predictive performance, as evidenced by an AUC reaching 0.88 [7]. Other groups used radiomics to predict the outcome and pseudoprogression of vestibular schwannoma treated with SRS [23,24]. Several studies explored radiomics to predict local control of brain metastases [25,26,27,28] or arteriovenous malformations [29,30,31] after SRS using radiomics.

Of the four sequences investigated, we found that T2w provided the best predictive performance for volumetric PA change (Table 2). This result agrees with our previous findings applying texture analysis to predict the volumetric outcomes of SRS in benign meningiomas and vestibular schwannomas. For benign meningiomas, we found that the histogram parameter *standard deviation* of voxel intensities of T2w images correlated best with volumetric change after SRS [17]. Increased T2w intensity values have been reported to relate to a soft consistency of meningiomas, increased vascularity, cellular atypia, and angioblastic or melanocytic components, as well as cystic degeneration and ischemic necrosis [32,33,34]. For vestibular schwannomas, the kurtosis of T2w image intensity values predicted progression best, with a sensitivity and specificity of 71% and 78%, while the minimum of the T2w voxel intensity values correlated significantly with the final regression of tumor volume per month [18].

Several studies investigated the use of MRI to preoperatively assess tumor consistency in PAs, as tumor consistency is a critical factor in surgical planning. Most existing studies support the ability of T2w to predict intraoperative consistency, while T1w has not been shown to offer any predictive value [35]. Hypointensity on T2w likely correlates with firmer tumors, possibly attributable to their increased collagen content and vascularity. In comparison, softer tumors tend to be hyperintense on T2w, which may relate to higher water content and/or cystic components [36,37,38]. Although multiple preoperative PA consistency assessment methods have been studied, none demonstrated sufficient accuracy and reliability in clinical use [39]. More recently, radiomics and machine learning-based models have achieved high precision and good AUC values [39]. Wan et al. developed a radiomics model built on combined T1w/CE-T1w/T2w images, with 11 imaging features exhibiting statistically significant differentiation between soft and hard PAs, providing an excellent performance with an AUC of 0.90 [40].

In functional PAs, in addition to volumetric tumor control, hormone remission is a mandatory treatment objective. As more genes must be silenced to achieve hormone remission, substantially higher PA margin doses are required. Because of higher margin doses, we observed a more favorable volumetric response in functional PAs (mean volume change/month: −2.21%) compared with nonfunctioning PAs (mean volume change/month: −1.22%). A preliminary analysis revealed enhanced associations within subdivided PA cohorts, achieving an AUC close to 1. Due to the small sample size of functional PA treatments, we were unable to separately perform a reliable statistical analysis in the subset of functional PA tumors. However, our findings warrant further investigation.

The high dimensionality of radiomics analysis is both its strength and its weakness, as its high dimensionality has been widely criticized. To address this issue, we pre-selected features by excluding those that were not significantly associated with the outcome, resulting in an extensive reduction in dimensionality. Additionally, our study showed that adding CP features to multi-sequence imaging data (CP + T1w + CE-T1w) did not improve predictive performance over single-sequence radiomics models.

## 5. Limitations

Although the patient group was highly homogeneous and vastly exceeded the sample size requirement, its size still posed a limitation. To enhance the clinical validity of the reported feature association with PAs’ volume response to GKRS, further studies in larger patient groups are warranted. The relatively short follow-up time and the retrospective design of the prognostic models were additional limitations. Moreover, predictive studies necessitate confirming the generalizability of the acquired findings through external validation, while internal prognostic validation in unseen test folds that were not part of the development cohort has already been carried out within this study. Therefore, further validation in external cohorts is needed to establish the prognostic clinical validity of the obtained predictive models. Additionally, despite the objective nature of the computational analysis technique, the workflow included residual subjectivity during the tumors’ VOI segmentation.

## 6. Conclusions

We demonstrated that a radiomics-based model using conventional MR imaging achieved excellent predictive classification and generalization performance, surpassing a model based on clinicopathological parameters. By achieving reliable predictions of the volumetric outcomes of SRS, radiomics might enable individualized treatment strategies, ultimately contributing to improved treatment outcomes for patients undergoing SRS for pituitary adenomas.

## Figures and Tables

**Table 1:** Patients, treatment characteristics, and treatment results.

	All PAs		Functional PAs		Nonfunctional PAs	
Patient and treatment characteristics	Value	Range	Value	Range	Value	Range
Number of patients	81	-	29 [36%]	-	52 [64%]	-
Age in years (mean, range)	45.6	(11.4/83.2)	38.3	(11.4/65.5)	49.7	(21.2/83.2)
Pre-SRS tumor volume in cm^3^ (mean, range)	6.30	(0.16/40.33)	6.00	(0.18/33.54)	6.39	(0.16/40.33)
Previous RT, SRS	0	-	0	-	0	-
Previous surgery	69 [85%]	-	25 [86%]	-	44 [85%]	-
KPS before SRS (mean, range)	83.3	(60/100)	82.7	(60/100)	83.8	(60/100)
Single fraction SRS treatments	49 [60%]	-	13 [45%]	-	36 [69%]	-
Hypofractionated SRS treatments	32 [40%]	-	16 [55%]	-	16 [31%]	-
Number of fractions (mean, range)	2.21	(1/5)	2.83	(1/5)	1.86	(1/4)
Gradient index (mean, range)	2.83	(2.42/3.60)	2.82	(2.48/3.48)	2.83	(2.42/3.60)
Coverage index (mean, range)	96.7%	(91.0%/100%)	96.8%	(91.0%/100%)	96.6%	(91.0%/100%)
Selectivity index (mean, range)	67.8%	(18.0%/89.0%)	63.6%	(18.0%/85.0%)	70.3%	(39.0%/89.0%)
Paddick conformity index (mean, range)	65.5%	(17.6%/83.7%)	61.5%	(17.6%/80.8%)	67.8%	(39.0%/83.7%)
Margin physical dose in Gy (mean, range)	20.5	(12/40)	26.7	(15/40)	17.0	(12/24)
Margin BED in Gy (mean, range)	100.9	(40.4/284.5)	106.2	(40.4/284.5)	97.8	(60.5/181.9)
Margin SFED in Gy (mean, range)	16.2	(11.1/35.0)	19.6	(11.8/35.0)	14.2	(11.1/20.0)
**Treatment results**	**Value**	**Range**	**Value**	**Range**	**Value**	**Range**
Follow-up period in months (mean, range)	40.4	(6.7/105.5)	38.6	(6.7/85.1)	41.4	(7.2/105.5)
Complete response	0 [0%]	-	0 [0%]	-	0 [0%]	-
Partial response (PR, decrease by ≥30%)	60 [74.1%]	-	27 [93%]	-	33 [63.5%]	-
Stable disease (SD, neither PR, no PD)	20 [24.7%]	-	2 [7%]	-	18 [34.6%]	-
Progressive disease (PD, increase by ≥20%)	1 [1.2%]	-	0 [0%]	-	1 [1.9%]	-
Absolute volume change in cm^3^ (mean, range)	−2.80	(−15.83/1.80)	−3.35	(−15.00/−0.06)	−2.49	(−15.83/1.80)
Relative volume change (mean, range)	−45.7%	(−90.2%/92.7%)	−55.4%	(−81.0%/−27.6%)	−40.2%	(−90.2%/92.7%)
Volume change per month (mean, range)	−1.58%	(−9.84%/1.75%)	−2.21%	(−9.84%/−0.57%)	−1.22%	(−4.83%/1.75%)

Abbreviations: PA, pituitary adenoma; SRS, stereotactic radiosurgery; RT, radiotherapy; KPS, Karnofsky Performance Status; BED, biologically effective dose; SFED, single-fraction equivalent dose.

**Table 2:** Association of models with continuous uncategorized PA volumetric response outcomes ^a^.

	Test Folds (10)	
Model	R^2^	Selected l
CP	0.272	0.0092
T1w	0.464	0.0086
CE-T1w	0.281	0.0130
T1w + CE-T1w	0.502	0.0144
CP + T1w + CE-T1w	0.584	0.0138
T2w	0.665	0.0115
FLAIR	0.312	0.0149

^a^ Linear LASSO regression was used to evaluate the association of models with the continuous, uncategorized tumor volume response outcome.

**Table 3:** The association of the clinical parameters and models with the outcome categorized by a cutoff at a −0.25% tumor volume change per natural logarithm of time.

Clinical Parameters				
*T*-Test in the Entire Cohort				
Feature ^a^	T-Statistic	*p*-Value	Mean ± SD	Mean Difference ± SD
Age	−3.38	0.001	45.5 ± 14.2	10.6 ± 3.0
Fraction number	2.62	0.011	2.2 ± 1.6	−0.80 ± 0.32
Dose per fraction	−0.06	0.953	13.1 ± 7.2	−0.26 ± 1.4
Accumulated dose	4.99	0.000	20.6 ± 6.7	−5.7 ± 1.2
BED	0.368	0.714	100.6 ± 44.6	−4.7 ± 9.1
SFED	2.588	0.012	16.2 ± 5.0	−2.5 ± 0.93
Coverage	0.711	0.479	96.7 ± 2.0	−0.32 ± 0.47
Selectivity	0.008	0.994	67.8 ± 11.6	0.37 ± 2.7
PCI	0.096	0.924	0.66 ± 0.11	0.001 ± 0.027
BOT	0.641	0.523	40.9 ± 24.6	−4.9 ± 5.2
**Chi-square test in the entire cohort**				
	**Pearson’s** **Chi-square**	***p*** **-value**	**Gamma**	***p*** **-value**
Functionality ^b^	0.42	0.001	0.81	<0.001
**Models ^c^**				
**AUC and accuracy in the test folds (8)** ^d^				
**Model**	**AUC**	**Accuracy**	**True positives**	**True negatives**
CP	0.846 ± 0.046	0.800 ± 0.049	0.542 ± 0.123	0.770 ± 0.049
T1w *	0.924 ± 0.022	0.859 ± 0.054	0.823 ± 0.049	0.850 ± 0.035
CE-T1w	0.759 ± 0.076	0.724 ± 0.091	0.614 ± 0.049	0.830 ± 0.113
T1w + CE-T1w *	0.899 ± 0.054	0.859 ± 0.062	0.810 ± 0.131	0.873 ± 0.089
CP + T1w + CE-T1w *	0.909 ± 0.016	0.854 ± 0.024	0.845 ± 0.051	0.866 ± 0.101

^a^ The independent sample *t*-test was used for variables with continuous values. Equality of variances was evaluated by Levene’s test. ^b^ The Chi-square test was used for the functionality variable because of its ordinal categorical values. ^c^ Models were constructed by combining the specified groups of features. ^d^ The test folds (30 patients) were reserved for the final evaluation to provide an unbiased assessment of the model’s performance on completely new, unseen data. Test folds were distinct from the validation folds used in the development cohort (51 patients). The presented AUC and accuracy values were obtained using an optimal classifier for each model. * Models that significantly differed from the benchmark CP model, according to an independent-samples *t*-test. Abbreviations: PCI, Paddick conformity index; BOT, beam on time; SFED, single-fraction equivalent dose; BED, biologically effective dose; CP, clinicopathological parameters.

**Table 4:** Prognostic performance of the indicated classifiers applied to the comprehensive CP + T1w + CE-T1w models ^a^.

Averages of Prognostic Evaluators ± SD				
	Training Folds (8)		Testing Folds (8)	
Classifier	AUC	Accuracy	AUC	Accuracy
Random forest	0.941 ± 0.033	0.867 ± 0.060	0.846 ± 0.048	0.773 ± 0.066
Naive Bayes	0.896 ± 0.052	0.804 ± 0.086	0.795 ± 0.073	0.704 ± 0.088
kNN	0.962 ± 0.028	0.892 ± 0.049	0.845 ± 0.056	0.790 ± 0.028
Logistic regression	0.991 ± 0.015	0.950 ± 0.042	0.877 ± 0.031	0.815 ± 0.064
Neural network	0.990 ± 0.020	0.957 ± 0.042	0.878 ± 0.033	0.824 ± 0.065
SVM	0.977 ± 0.017	0.927 ± 0.045	0.889 ± 0.043	0.820 ± 0.045

^a^ LASSO selected eight features from each randomly chosen development fold (training and validation folds), including 51 patients, followed by classification using the specified classifiers. Subsequently, the models were assessed on the separate, unseen test folds consisting of 30 patients. Abbreviations: SVM, support vector machines; kNN, k-nearest neighbors.

**Table 5:** Feature composition of the prognostically best performing model incorporating the CP + T1w + CE-T1w features ^a^.

Feature	B	95% CI		P
Age	1.192	−18.8	122.6	0.015
Accumulated dose	−1.020	−204.3	13.4	0.084
Orig_firstorder_Entropy_T1w	−5.144	−989.7	−3.30	0.002
Log_gldm_Smalldepemph_T1w	1.775	−84.4	417.5	0.026
Log_glcm_Id_T1w	−2.936	−601.5	8.9	0.010
Lbp2D_gldm_lgdepLowGraylevemph_T1w	−0.784	−147.4	400.8	0.296
Logarithm_glcm_JointEnergy_CE-T1w	0.681	−33.3	111.3	0.040
Lbp2D_glrlm_LongRunLowGraylevemph_T1w	0.955	−218.6	228.8	0.257

^a^ This model was computed against the 0.25% PA volumetric response per logarithmic month. Abbreviations: Orig, original; Dep, dependence; Emph, emphasis; Lg, large; Lev, level.

## Data Availability

The datasets generated during and analyzed during the current study are available from the corresponding author on reasonable request.
